# Assessment of the diagnostic accuracy and relevance of a novel ELISA system developed for seroepidemiologic surveys of *Helicobacter pylori* infection in African settings

**DOI:** 10.1371/journal.pntd.0009763

**Published:** 2021-09-09

**Authors:** Evariste Tshibangu-Kabamba, Bui Hoang Phuc, Vo Phuoc Tuan, Kartika Afrida Fauzia, Augustin Kabongo-Tshibaka, Nadine Kalenda Kayiba, Angel Rosas-Aguirre, Brecht Devleesschauwer, Alain Cimuanga-Mukanya, Patrick de Jésus Ngoma Kisoko, Takashi Matsumoto, Junko Akada, Ghislain Tumba Disashi, Dieudonné Mumba Ngoyi, Yasutoshi Kido, Niko Speybroeck, Yoshio Yamaoka

**Affiliations:** 1 Department of Environmental and Preventive Medicine, Faculty of Medicine, Oita University, Oita, Japan; 2 Department of Internal Medicine, Faculty of Medicine, University of Mbujimayi, Mbujimayi, DR Congo; 3 Research Center for Infectious Diseases Sciences & Department of Parasitology, Graduate School of Medicine, Osaka City University, Osaka, Japan; 4 Department of Endoscopy, Cho Ray Hospital, Cho Ray, Vietnam; 5 Institute of Tropical Disease, Universitas Airlangga, Surabaya, Indonesia; 6 Department of Public Health and Preventive Medicine, Faculty of Medicine, Universitas Airlangga, Surabaya, Indonesia; 7 Research Institute of Health and Society (IRSS), Université catholoique de Louvain, Brussels, Belgium; 8 Department of Public Health, Faculty of Medicine, University of Mbujimayi, Mbujimayi, DR Congo; 9 Department of Epidemiology and Public Health, Sciensano, Brussels, Belgium; 10 Department of Veterinary Public Health and Food Safety, Ghent University, Merelbeke, Belgium; 11 Department of Gastroenterology and Hepatology, Faculty of Medicine, University of Kinshasa, Kinshasa, DR Congo; 12 Department of Gastroenterology and Hepatology, General Referential Hospital of Bukavu, Bukavu, DR Congo; 13 Department of Parasitology, National Institute of Biomedical Research, Kinshasa, DR Congo; 14 Department of Medicine, Gastroenterology section, Baylor College of Medicine, Houston, Texas, United States of America; 15 Global Oita Medical Advanced Research Center for Health, Oita University, Yufu, Japan; NIH-National Institute for Research in Tuberculosis-ICER, INDIA

## Abstract

Beside diagnostic uncertainties due to the lack of a perfect gold standard test for *Helicobacter pylori* infection, the diagnosis and the prevalence estimation for this infection encounter particular challenges in Africa including limited diagnostic tools and specific genetic background. We developed and evaluated the accuracy of an enzyme-linked immunosorbent assay (ELISA) system tailored for *H*. *pylori* genetics in Africa (HpAfr-ELISA). Strains belonging to main genetic populations infecting Africans were exploited as sources for whole-cell antigens to establish in-house the ELISA system. A phase II unmatched case-control study explored the diagnostic accuracy of the HpAfr-ELISA using a training set of samples collected from dyspeptic patients from Kinshasa, the Democratic Republic of Congo (DRC) who had been tested with invasive standard tests (i.e., histology, culture, and rapid urease test) in 2017. Then the assay was cross-validated through a community-based survey assessing the prevalence of *H*. *pylori* and associated factors in 425 adults from Mbujimayi, DRC in 2018. Bayesian inferences were used to deal with statistical uncertainties of estimates (true prevalence, sensitivity, and specificity) in the study population. At its optimal cut-off-value 20.2 U/mL, the assay achieved an estimated sensitivity of 97.6% (95% credible interval [95%CrI]: 89.2; 99.9%) and specificity of 90.5% (95%CrI: 78.6; 98.5). Consistent outcomes obtained at repeated tests attested the robustness of the assay (negative and positive agreements always > 70%). The true prevalence of *H*. *pylori* was estimated 53.8% [95%CrI: 42.8; 62.7%]. Increasing age (adjusted odds ratio [aOR] > 1.0 [95% confidence interval (CI): > 1.0; 1.1]; p<0.001), overcrowding households (aOR = 3.2 [95%CI: 2.0; 5.1]; p<0.001), and non-optimal hand hygiene (aOR = 4.5 [95%CI: 2.0; 11.4]; p = 0.001) were independently associated with the *H*. *pylori*-seropositivity. The novel ELISA system has demonstrated good diagnostic accuracy and potential usefulness for management and mitigation strategies for *H*. *pylori* infection in African settings.

## Author summary

Enzyme-linked immunosorbent assay (ELISA) systems are useful tools for mass screening of *Helicobacter pylori* infection but their use encounters important challenges in Africa. This is mainly due to the specific genetic background of African *H*. *pylori* that may jeopardize the accuracy of currently available ELISA kits. In this study, we developed a new ELISA system, i.e. HpAfr-ELISA, based on pooled antigens of *H*. *pylori* that represent main genetic populations colonizing Africa. This new assay could be validated analytically using training samples collected in *H*. *pylori* positive and negative patients from the Democratic Republic of Congo (DRC). Then, the tool was successfully applied to a real-world community-based mass survey aiming at assessing the epidemiology of *H*. *pylori* in a rural area of the DRC. The characteristics of the HpAfr-ELISA assay indicated good robustness and high performances for detection of *H*. *pylori*. Statistical modeling enabled estimating the true latent prevalence of *H*. *pylori* infection and identifying factors predicting the exposition to the infection in the study population. This outcome will be useful for drawing efficient health policies and optimally allocating available resources to fight the *H*. *pylori* infection and related complications (e.g., gastric cancer) in the study area and globally in Africa.

## Introduction

*Helicobacter pylori* is a Gram-negative, spiral shaped, flagellated *microaerophilic* bacterium that infects half of living humans worldwide [1]. This infection is the major cause of a wide spectrum of diseases including non-immune chronic gastritis, peptic ulcer diseases, and gastric cancer [1,2]. Several risk factors play an important role in *H*. *pylori* infection including gender, age, and low socioeconomic status [3]. However, a substantial epidemiological variation and an exceptionally wide genetic diversification of this infection exist in different regions and human populations [1,3,4]. Strategies for effectively addressing this public health issue including the individual diagnosis and mass screening should thus be adjusted to population background.

*H*. *pylori* infection is diagnosed by several methods including invasive (e.g., culture from gastric biopsies, histology, and rapid urease assay) and noninvasive tests (e.g., PCR on stool samples, urease breath test, rapid urine test, serological test, and stool antigen test) [5–7]. Each of these tests carries proper imperfections and advantages but none of them fulfills the statistical requirements for perfect a gold standard test which is a reference and error-free diagnostic test [6,7]. Consequently, all tests have a potential for misclassifications of infected and non-infected people that can lead to inappropriate clinical and public health decisions. Unlike invasive tests, noninvasive ones are widely used for epidemiological investigations [7]. Serological tests such as Enzyme-linked immunosorbent assays (ELISA) are the only diagnostic methods that are able to detect past exposure to *H*. *pylori* [8]. Basically, the ELISA technique is relatively simple, reliable, fast, inexpensive, and has interesting high-throughput capabilities [5,6,8]. Several ELISA kits for *H*. *pylori* infection are commercially available [9]. For all these reasons, this technique is highly attractive for screening of large communities to evaluate prevalence including within regions with limited resources. However, the ELISA for *H*. *pylori* needs adjustment for specific regions and populations as infections in varied areas have clearly indicated different immunogenic properties [9–13]. Moreover, the nature of antigens from the same isolates plays a significant role in the improvement of ELISA sensitivity and specificity [14]. *H*. *pylori* species displays a diverse protein profile (e.g., CagA, VacA, UreaB, OipA, Heat shock protein B, Flagellar proteins) with more or less enhanced antigenic properties which are putative components of vaccines or ELISA systems [15–18]. During the colonization of the human stomach by *H*. *pylori*, these antigens activate humoral and cell mediated immune response with elevated titers of serum immunoglobulin predominantly of IgG class [15,17]. Whole-cell antigens covering a broad range of bacterial antigens have been found to be more immunogenic than isolated antigenic components [17].

Otherwise, compared to other continents, Africa has recorded the highest prevalence of *H*. *pylori* infection; however the epidemiology of this infection remains relatively less explored at community levels [3,4,19]. Africa is colonized by *H*. *pylori* bacterial strains that are the most genetically distant and structurally divergent from remaining species populations [20,21]. Beyond diagnostic uncertainties due to the lack of a perfect gold standard test, the clinical diagnosis and the prevalence estimation of *H*. *pylori* are surrounded with significant operational challenges in Africa. These include restricted access to diagnostic tools such as gastrointestinal endoscopy and *H*. *pylori* culture facilities, limited number of trained operators, specific genetic background, and huge favoring conditions for infection transmission [19]. Regarding ELISA systems, it is likely that available commercial kits that have essentially been optimized and evaluated in developed countries may not be suitable for Africa. We thus developed in-house a novel ELISA system (i.e., HpAfr-ELISA) based on *H*. *pylori* whole-cell antigens retrieved from bacterial isolates representing the main genetic populations infecting humans in Africa. The goal was to provide a serodiagnostic and seroepidemiological tool for *H*. *pylori* infection in Africa. This study aimed to determine the diagnostic accuracy and robustness of this tool in compliance with the evidence-based architecture of diagnostic research [22]. The diagnostic relevance of this system was further explored during a real-world community-based survey conducted for estimating the prevalence of *H*. *pylori* infection and for identifying its most predictive factors in the Democratic Republic of Congo (DRC), Middle Africa.

## Methods

This study is reported following the “Strengthening the Reporting of Observational studies in Epidemiology” (STROBE) statement guidelines [23] and the STROBE checklist is provided in supporting information (**S1 Table**).

### Ethics statement

All study subjects provided their informed consent for inclusion before participation in the study. A formal consent was thus obtained written. The study was conducted in accordance with the Declaration of Helsinki, and the protocol was approved by the Ethical Review Boards of the University of Mbujimayi in DRC (N#33/MREC/UM/ETK/GDT/2018).

### Flowchart for the study methodology

**Fig 1:**
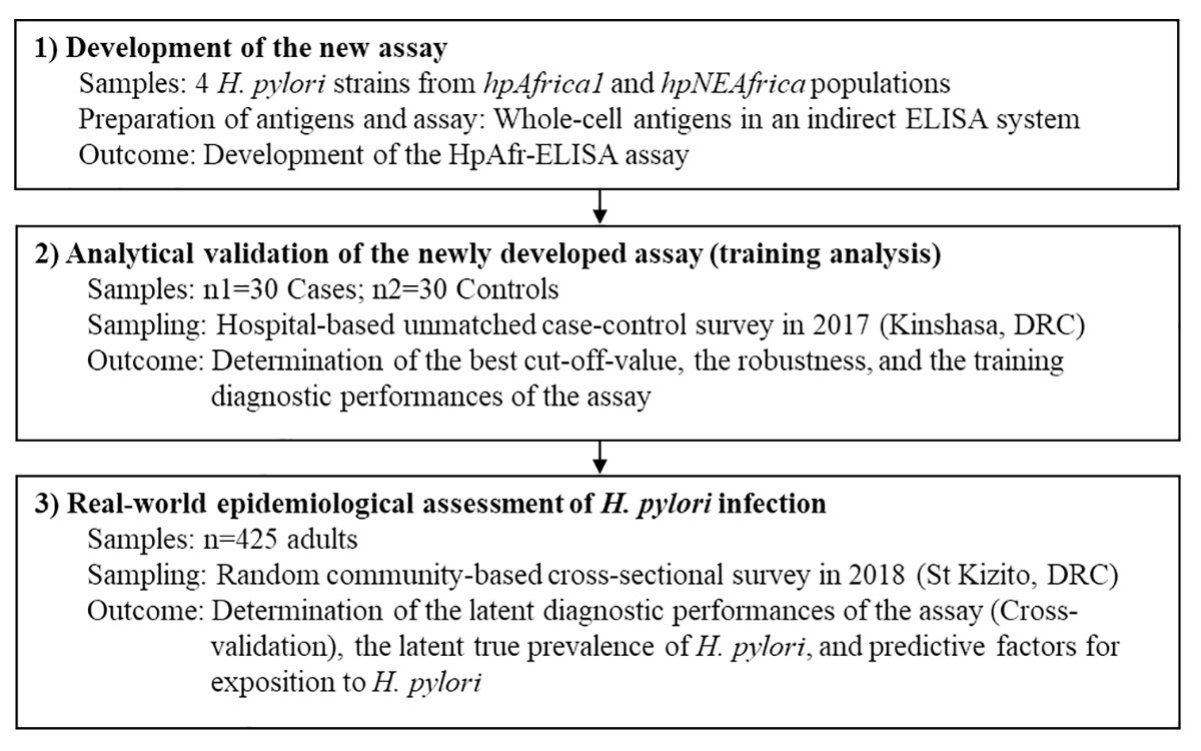
Flowchart for the study methodology. This study followed three main steps including the development of the new ELISA assay followed by its analytical validation using a training set of clinical samples (Cases and Controls), and, finally, its implementation during a real-world epidemiological survey.

The flowchart in **Fig 1** displays the main methodological steps of this study.

### Study area, population and design

This is a phase II evaluation of a newly developed diagnostic test. First, the diagnostic accuracy of the test was evaluated experimentally through an unmatched case-control analysis performed using a training set of equal numbers of *H*. *pylori*-positive cases (n = 30) and *H*. *pylori*-negative controls (n = 30) obtained during a hospital-based survey on dyspeptic patients who underwent upper gastrointestinal examination by endoscopy in 2017 in Kinshasa, the capital city of The Democratic Republic of Congo (DRC) [24]. This sample size was expected to be a priori sufficient for reliably detecting an **Area Under the Curve (AUC)** of ≥ 0.70 as statistically different from 0.50 with an 80% power (α = 0.05; 2-sided test) [25]. Then, the test accuracy was cross-validated using samples (cross-validation set) from a mass community-based survey carried out in August 2018 in the Health Area of Kizito (coordinates: latitude, 6°6′S to 6°7′S; and longitudes, 23°37′E to 25°38′E) that is located on the outskirts of Mbujimayi, in the center of the DRC. This cross-sectional survey recruited participants at household level. Targeted households were randomly sampled using a frame elaborated based on satellite images captured over the study area with Google Earth (www.google.com/intl/eng/earth). Inclusion criteria were that the respondent was an adult (≥18 years old) residing in the study area and who provided an informed consent. Only one or two potentially eligible participants could be recruited from each household on a first-come, first-included basis. Exclusion criteria comprised those from whom a good quality biological sample or a full interview could not be obtained among potentially eligible subjects. A minimum sample size of 384 participants was deemed sufficient for this survey (based on a worldwide expected prevalence of 50% [4] with a precision of 5% and a type I error of 5%).

### Data collection and definition of variables

A piloted standard questionnaire was used to collect socio-demographic information and dyspepsia symptoms in each study participants. Dried blood spots (DBS) were sampled using Whatman 903 filter card (GE Healthcare Ltd., UK) for biological testing. The variable of interest was the level of anti*-H*. *pylori* IgG. Participants were considered as being *H*. *pylori*-seronegative or -seropositive respectively when the anti*-H*. *pylori* IgG level was ≤ or > the best cut-off-value of the assay. Types of housing (i.e., structure of walls, floor, and roof) were used to generate a wealth and ownership categorization by Principal Component Analyses (PCA) rating participants into 2 socioeconomic levels: “more economically disadvantaged” and “less economically disadvantaged”. An index reflecting the crowding of residences was defined based on the number of household members sharing the same sleeping room. “Overcrowded households” were thus considered as those with an index of occupancy higher than the median index in the study population. The level of hand hygiene was appreciated as optimal or non-optimal based on the reported frequency of hand washing after being to toilets (i.e., corresponding to “always” or to one of the following “occasionally”, “sometimes”, “frequently”, and “usually”). Dyspepsia symptoms and their severity were evaluated using three clinical scores as established in previous studies: i) the Dyspepsia Symptom Severity Index (DSSI) with its three categories of symptoms referred to as “Dysmotility-like”, “Reflux-like”, and “Ulcer-like” [26]; ii) the Glasgow Dyspepsia Severity Score (GDSS) [27]; and iii) the Global Overall Symptom (GOS) Scale that assess the impact of symptoms on people’s quality of life [28]. The dyspepsia syndrome was defined based on the Rome IV criteria as any combination of 4 symptoms: postprandial fullness, early satiety, epigastric pain, and epigastric burning that are severe enough to interfere with the usual activities and occur at least 3 days per week over the last 3 months with an onset of at least 6 months in advance [29].

### DBS samples processing

DBS samples were processed following the protocol developed by Grüner et al. [30]. Briefly, through a skin puncture made on a finger, five drops of blood were gently transferred to each of the circles of a Whatman 903 filter card. The filter card was put upon collection on a clean paper towel in a biohazard safety box to be dried at ambient temperature for up to 4 hours. Then, a single gas-impermeable zipper bag with a desiccant was used for storage of each filter card at ambient temperature. All DBS samples were transported to Oita University in Japan for laboratory analyses. Before testing, DBS were punched out from cards, transferred to a 24-well plate and eluted overnight on a shaker using 500 μL solution of phosphate-buffered saline (PBS, pH 7.2–4, Wako, Japan) containing 0.05% Tween 20 and 0.08% sodium azide. Eluted DBS were used for HpAfr-ELISA testing of *H*. *pylori*.

### Development of the HpAfr-ELISA and laboratory testing

Anti*-H*. *pylori* IgG antibodies were measured using the HpAfr-ELISA assay, an indirect ELISA system developed in-house. This ELISA system immobilizes antigens from whole cells of four representative *H*. *pylori* strains isolated from Congolese patients in 2017 [24]. These isolates were selected based on their genetic background as they clustered in *hpAfrica1* (two strains) and *hpNEAfrica* (two strains) (**S1 Fig**)–the two main genetic populations of *H*. *pylori* species colonizing the Sub-Saharan Africa [21,31,32]. To develop this assay, pure bacterial cells of each strain were suspended well in PBS and treated with 1% octyl-glucoside and 1mM Phenylmethylsulfonyl fluoride (PMSF) protease inhibitor (ThermoFisher Scientific, USA) in PBS. Then, 2,990 μL of each whole cell antigens lysate solution was dialyzed for 12 hours at 4°C using A-Lyzer 10K Dialysis Cassettes G2 (ThermoFisher Scientific, USA) in 2L of PBS. Using the BCA microassay (TaKaRa bio, Japan) with bovine serum albumin (BSA) as standard, the antigens concentration was determined in 10 μL of each lysate solution before and after dialysis. An equimolar mixture of antigen solutions extracted from each *H*. *pylori* isolate was prepared and used to coat a 96-wells Maxisorp ELISA plate (Immobilizer-Amino Plate, UK). Each well was thus coated with 100 μg of antigens mixture in 100 mM Na_2_CO_3_-NaHCO_3_ buffer (pH 9.6) overnight and washed three times with PBS containing 0.1% Tween 20 (PBS-0.1T). After blocking ELISA plates with 0.2% BSA in PBS, 1:1,000 eluted DBS samples were reacted for 30 minutes. A solution of 1:80,000 peroxidase-conjugated secondary anti-human IgG antibodies (Jackson Immuno Research Labs, USA) was added for 30 more minutes to each reacting well after washing the plates with PBS-0.1T. The final detection was performed with POD/TMB substrate (Nacalai Tesque, Inc., Japan). This ELISA system was optimized and calibrated using the training set of blood samples from Congolese patients whose *H*. *pylori* status was already established using culture, histology or rapid urease test applied as previously described (**S2 Table**) [31,33,34]. Polyclonal rabbit anti-*Helicobacter pylori* IgG (Dako, Danmark) with detection by 1:80,000 peroxidase-conjugated secondary anti-rabbit IgG antibodies (Jackson Immuno Research Laboratories, Inc; USA) was used as an internal positive control while water, PBS and carbonate buffer were set as negative controls during all reactions. We defined that 1 U/mL anti-*H*. *pylori* antibodies from human was comparable to 1ng/mL anti-*H*. *pylori* rabbit IgG.

### Data analysis

The data were entered into Microsoft Excel spreadsheets (Microsoft Corp., Redmond, WA, USA, 2010) and analyzed using R software version 4.0.2 [35]. Data manipulations were performed with the *tidyverse* package [36]. Principal Component Analyses (PCA) were conducted using *FactoMineR* and *Factoextra* packages [37,38]. The AUC and ROC (**Receiver Operating Characteristics**) curves were estimated, analyzed, compared, and applied to determine the best cut-off-value of the assay using *pROC* package [39]. The DeLong’s test was used to evaluate the difference in AUCs of two correlated ROC curves. Qualitative variables were compared between *H*. *pylori-*seropositive and–seronegative groups using the Chi-square test or the Fisher’s exact test when appropriate. In contrast, quantitative variables were compared using parametric the Wilcoxon rank sum test with continuity correction after testing non-parametric conditions with the Shapiro-Wilk test and the Levene test. The reliability of the ELISA was assessed on both the training and the cross-validation sets through the Cohen’s Kappa and the Kendall’s W statistics calculated with *KappaGUI* and *synchrony* packages [40,41]. The 95% confidence interval (95%-CI) of proportions was estimated based on Clopper-Pearson exact interval. Uni- and multivariable logistic regression models were fitted to assess the association between different factors and the *H*. *pylori*-seropositivity. The final multivariable model was selected based on Akaike’s Information Criterion (AIC) values estimated for different intermediates models using both a backward and forward approach. For all the statistical tests applied, the value of p<0.05 was considered as the statistical significance threshold. Bayesian methods were applied for inferring latent estimates (e.g., true prevalence, sensitivity, and specificity) in the study population as updated posterior probabilities sampled through predefined probability distributions and Markov Chain Monte Carlo (MCMC) simulations [42–48]. The Bayesian model was fitted using *prevalence* package [44]. The median of each parameter was used to describe the uncertainty with a 95% credible interval (95%-CrI) based on the **Highest Density Interval (HDI).** At four parallel MCMC chains, each for 10,000 iterations for all the models with a burn-in period of 10,000 iterations, were performed. The parameters of each individual model were adjusted until the convergence of MCMC runs could be attested by Gelman and Rubin’s diagnostics measure (close to 1) [49]. The details of the Bayesian model fitted are presented in **S1 File**.

## Results

### Performance and robustness of HpAfr-ELISA

**Fig 2:**
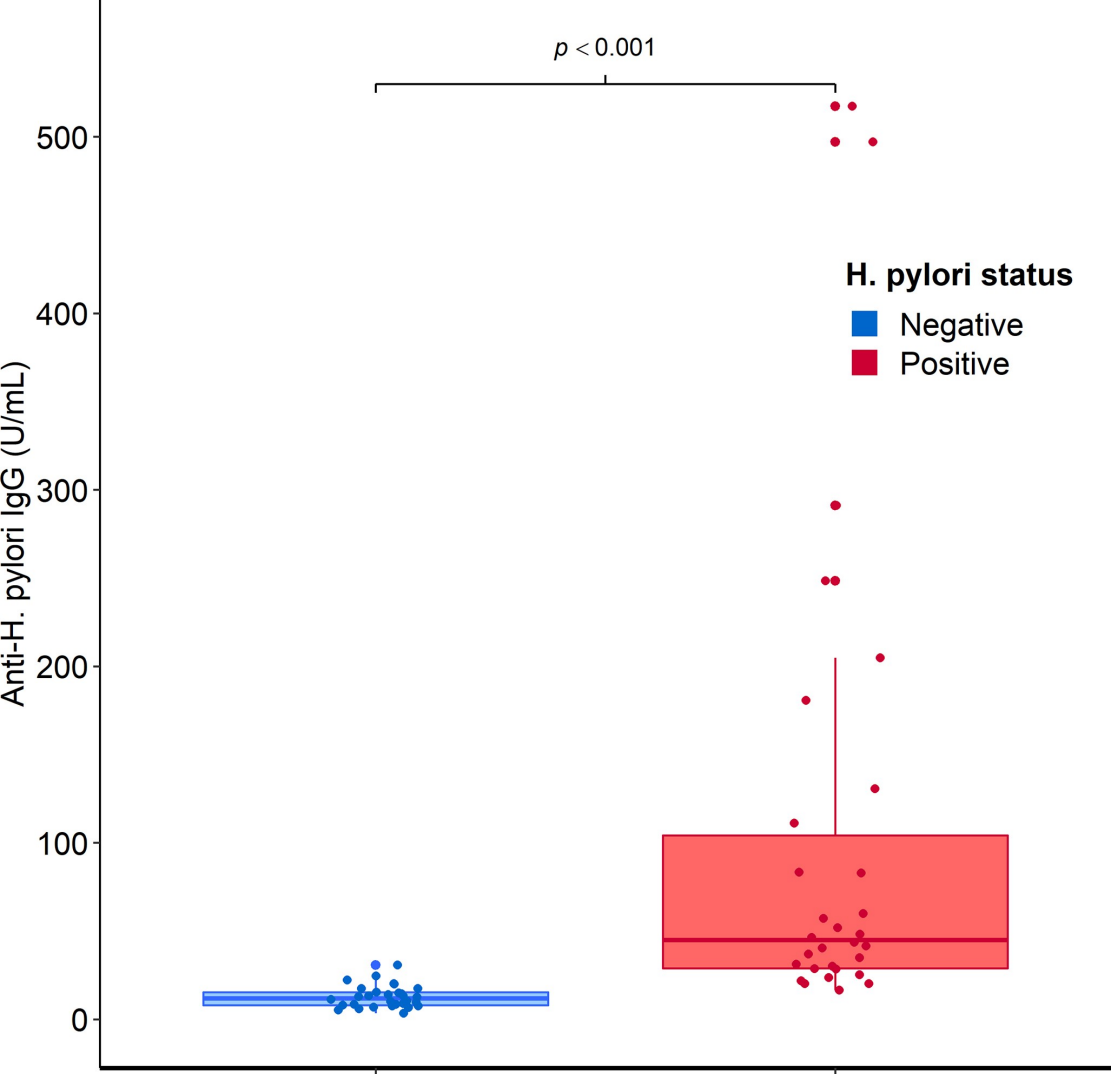
Comparison of the level of anti-*H*. *pylori* IgG antibodies between *H*. *pylori* negative and positive samples. This figure compares the rates of anti-*H*. *pylori* IgG antibodies between *H*. *pylori* negative and positive patients in the training set whose status had been established by standard invasive tests (i.e., histology, culture, and rapid urease test). Hence, medians of antibodies rates with interquartile intervals (IQR) were 45.0 (IQR: 75.2) and 11.9 (IQR: 7.3) U/mL respectively in *H*. *pylori* positive and negative patients. The difference of anti-*H*. *pylori* IgG levels between the two groups of patients was statistically significant (p = <0.001).

**Fig 3:**
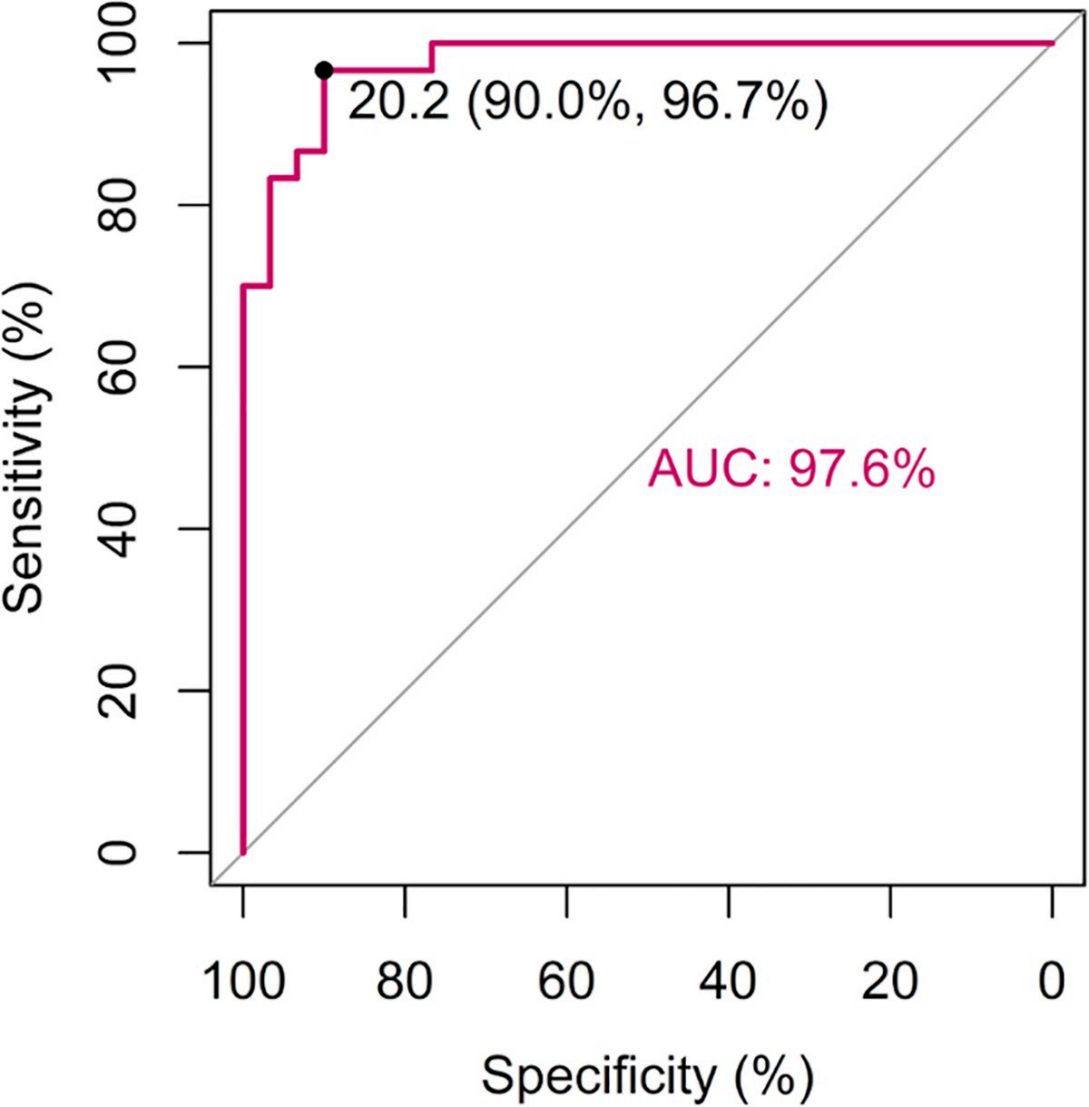
ROC/AUC and best cut-of-value of the HpAfr-ELISA assay in the training set. The plot displayed on this figure represent the ROC (**receiver operating characteristic) and the AUC (**Area under the *ROC Curve) obtained with HpAfr-ELISA on the training set using the H*. *pylori status by standard invasive tests (i*.*e*., *histology*, *culture*, *and rapid urease test) as reference*. This plot indicates that the ROC curve of HpAfr-ELISA had an AUC of 97.6% with its best cut-of-value being 20.2 U/mL of anti*-H*. *pylori* IgG.

**Fig 4:**
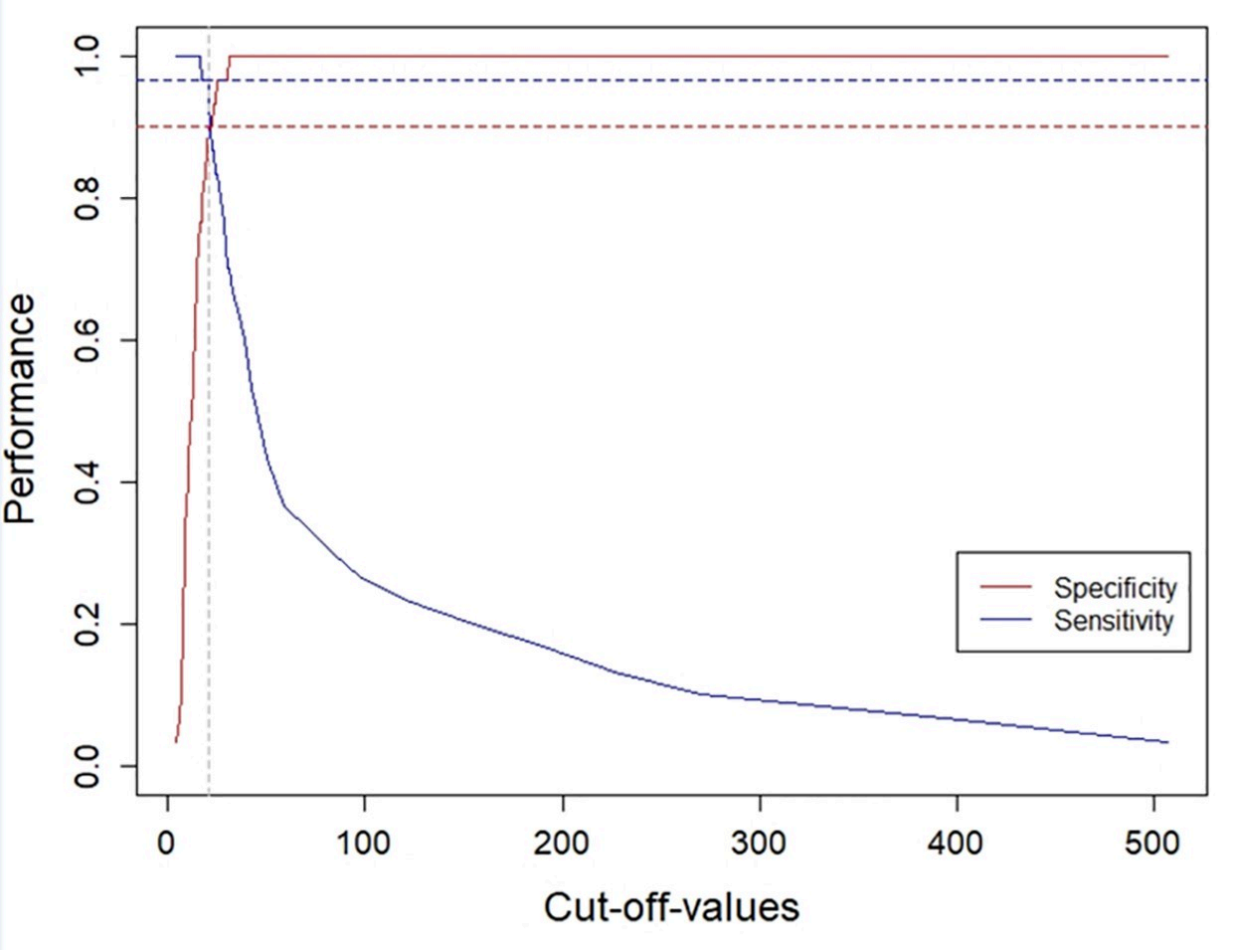
Sensitivity and specificity of the HpAfr-ELISA assay in the training set. This plot indicates that the HpAfr-ELISA assay had a sensitivity of 96.7% (dashed blue line) and a specificity of 90.0% (dashed red line) when considering its best cut-off-value (dashed black line) defined at the Youden index of the dataset (the point with the maximum specificity + sensitivity).

**Fig 5:**
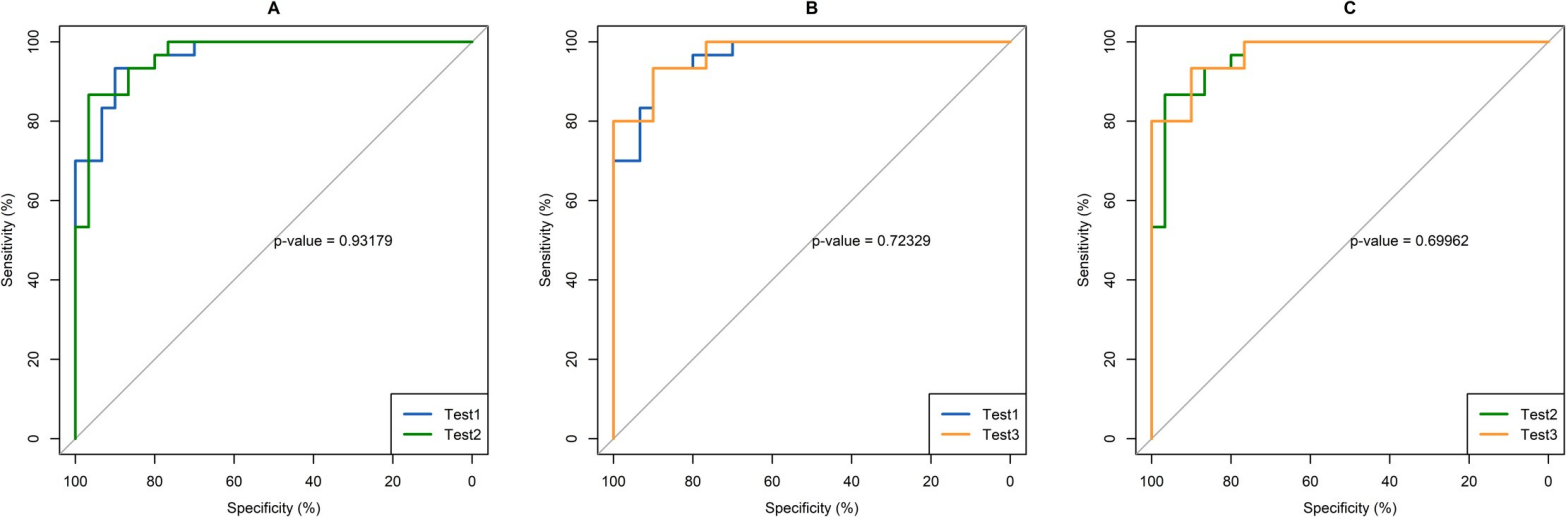
Comparison of ROCs/AUCs of the HpAfr-ELISA assay at three repeated testing. Plots displayed on the figure represent ROC curves and AUCs obtained at repeated tests (test 1, test 2, and test 3) on the training set in intra-observer. No significant difference between the ROC curve was noted (p>0.05).

Average levels of anti-*H*. *pylori* IgG antibodies obtained from repeated testing (three times) on the training set with the HpAfr-ELISA were determined for individual participants. Hence, samples from *H*. *pylori* positive patients (i.e., based on standard invasive tests) had significantly higher anti-*H*. *pylori* IgG levels than those from negative ones (45.0 interquartile range [IQR]: 75.2) and 11.9 (IQR: 7.3) U/mL; p = <0.001) (**Fig 2**). The ROC curve displayed an AUC of 97.6% climaxing at its best cut-of-value 20.2 U/mL of anti-*H*. *pylori* IgG with corresponding sensitivity 96.7% and specificity 90.0% (**Fig 3; Fig 4**). To assess the robustness of the assay, we considered individual outcomes at repeated testing of both the training set and a larger dataset from a community-based survey (cross-validation set). Repeated training tests thus indicated no significance difference in the ROC curve (p>0.05) (**Fig 5**). Furthermore, based on the best cut-off-value of the ROC/AUC, the ELISA indicated high indices for qualitative agreement of the outcomes during repeated tests on both the training samples (Kappa = 0.833; PA = 0.915; NA = 0.918) and the cross-validation sets (Kappa = 0.789; PA = 0.881; NA = 0.908) (**Table 1**). Likewise, significantly high indices for quantitative agreement were noted with both sets (W = 0.956 to 0.981; p = 0.001 and W = 0.631; p = 0.001) (**Table 2**). As part of cross-validation, we assessed the performance of the assay on the dataset from the community-based survey but in the absence of a perfect gold standard test. Therefore, stochastic simulations under Bayesian assumptions were performed with prior sensitivity and specificity defined as Beta distribution of results obtained on the training set (**S1 File**). The latent sensitivity and specificity of HpAfr-ELISA were thus respectively estimated 97.6% with a 95% credible interval (95%CrI) from 89.2 to 99.9%, and 90.5% with 95%CrI from 78.6 to 98.5 in the study population (**Table 3**).

**Table 1:** Qualitative agreement of HpAfr-ELISA outcomes at two repeated testing.

HpAfr-ELISA testing			Test 2		Kappa	PA	NA	p-value
			Positive	Negative				
**Training set**	**Test 1**	**Positive**	28	3	0.833	0.915	0.918	<0.001
		**Negative**	2	27				
**Cross-validation set**	**Test 1**	**Positive**	218	20	0.789	0.881	0.908	<0.001
		**Negative**	24	163				

(*) Kappa: Coefficient of Cohen’s Kappa; PA: Positive agreement index; NA: Negative agreement index

**Table 2:** Quantitative agreement of HpAfr-ELISA outcomes at two repeated testing.

HpAfr-ELISA testing		n	Anti-*H*. *pylori* IgG rates (U/mL)			W	S	p-value
			Median (IQR)	Max	Min			
**Training set**	**Test 1**	60	23.1 (38.4)	583.5	1.21	0.981	0.963	0.001
	**Test 2**	60	20.4 (30.1)	475.7	3.1			
**Training set**	**Test 3**	60	21.1 (29.9)	492.7	5.7	0.974	0.949	0.001
	**Test 2**	60	20.4 (30.1)	475.7	3.1			
**Training set**	**Test 1**	60	23.1 (38.4)	583.5	1.21	0.956	0.912	0.001
	**Test 3**	60	21.1 (29.9)	492.7	5.7			
**Cross-validation set**	**Test 1**	425	22.9 (30.4)	495.1	3.3	0.631	0.617	0.001
	**Test 2**	425	23.3 (26.5)	432.2	3.1			

(*) W: Coefficient of Kendall’s W corrected for tied ranks; S: Coefficient of Spearman’s ranked correlation; p-value: p-value of Kendall’s W

**Table 3:** Bayesian estimation of the latent diagnostic performance of HpAfr-ELISA in the absence of a perfect gold standard test.

Parameter	Counts	Training performance at the best cut-off-value, % [95%CI]	Latent performance estimate in the population, Median % [95%CrI]
Sensitivity	29/30	96.7 [82.8; 99.9]	97.6 [89.2; 99.9]
Specificity	27/30	90.0 [73.5; 97.9]	90.5 [78.6; 98.5]

(*) 95%CI: 95% Confidence Interval; 95%CrI: 95% Credible Interval

### Epidemiology of the *H*. *pylori* infection in the study population

The community-based survey included 425 participants with a median age 29 years old (IQR: 25) and comprising 59.8% of female subjects. Anti-*H*. *pylori* IgG concentrations ranged from 3.4 to 463.7 U/mL and in total, 241 out of 425 participants (56.7%) were *H*. *pylori*-seropositive based on the cut-off-value of 20.2 U/mL. Considering prior information related to the ranges of sensitivity and specificity obtained from the training experiment (**Table 3**), the true prevalence of *H*. *pylori* infection in the study population was estimated 53.8% with 95%CrI lying between 42.9 and 62.7% (**Table 4**). Baseline characteristics of study subjects indicated that *H*. *pylori*-seropositive participants were older than seronegative ones (35.5 (IQR: 36) and 26 (IQR: 16) years old; p<0.001). In addition, *H*. *pylori*-seropositive participants comprised significantly higher proportions of individuals who had been married than single (p = 0.043), who were holding a regular profession (p = 0.003), or who were recruited from overcrowded households (p<0.001). Furthermore, a significantly smaller proportion of individuals who had an optimal level of hand hygiene (i.e., reportedly washing hands “always” after being to toilets) were *H*. *pylori*-seropositive compared to those who had non-optimal hand hygiene (i.e., who were washing hands “occasionally” to “usually”) (3.3% and 96.7%; p<0.001) (**Table 5**).

**Table 4:** Bayesian estimation of the latent true prevalence of the *H*. *pylori* infection and the dyspeptic syndrome in the study population.

Parameter	Counts	Apparent prevalence, % [95%CI]	Latent true prevalence estimate, Median % [95%CrI]
*H*. *pylori* infection	241/425	56.7 [51.8; 61.5]	53.8 [42.8; 62.7]

(*) 95%CI: 95% Confidence Interval; 95%CrI: 95% Credible Interval

**Table 5:** Baseline socio-demographic characteristics of study participants (n = 425).

Characteristics	Anti-*H*. *pylori* serology		Total	p-value
	Seropositive	Seronegative		
	n (%) or median (IQR)	n (%) or median (IQR)	n (%) or median (IQR)	
**Gender**				
Female	150 (62.2)	104 (56.5)	254 (59.8)	
Male	91 (37.8)	80 (43.5)	171 (40.2)	0.272
**Age (years)**				
Median age (IQR)	35.5 (36)	26 (16)	29 (25)	<0.001
**Marital status**				
Married	185 (76.8)	125 (67.9)	310 (72.9)	
Single	56 (23.2)	59 (32.1)	115 (27.1)	0.055
**Religion**				
Christian	233 (96.7)	177 (96.2)	410 (96.5)	
Non-Christian	8 (3.3)	7 (3.8)	15 (3.5)	0.998
**Education level**				
Elementary	56 (23.2)	40 (21.7)	96 (22.6)	Ref
College/University	18 (7.5)	15 (8.2)	33 (7.8)	0.860
High school	167 (69.3)	129 (70.1)	296 (69.6)	0.833
**Holding a regular profession**				
Yes	135 (56.0)	76 (41.3)	211 (49.6)	
No	106 (44.0)	108 (58.7)	214 (50.4)	0.004
**Crowding of households**				
Less crowded household	99 (41.1)	128 (69.6)	227 (53.4)	
Overcrowded household	142 (58.9)	56 (30.4)	198 (46.6)	<0.001
**Duration of residence in the area (years)**				
Median duration (IQR)	4 (11)	4 (11.3)	4 (11)	0.493
**Socioeconomic category**				
Category 1: "Most economically disadvantaged"	129 (53.5)	97 (52.7)	226 (53.2)	
Category 2: "Less economically disadvantaged"	112 (46.5)	87 (47.3)	199 (46.8)	0.946
**Sources for daily drinking water**				
Well water	3 (1.2)	1 (0.5)	4 (0.9)	
Tap water	238 (98.8)	183 (99.5)	421 (99.1)	0.637
**Type of toilet facilities**				
Private toilets	37 (15.4)	24 (13.0)	61 (14.4)	
Toilets shared between several families	204 (84.6)	160 (87.0)	364 (85.6)	0.594
**Quality of hand hygiene**				
Non-optimal hand hygiene	233 (96.7)	147 (79.9)	380 (89.4)	
Optimal hand hygiene	8 (3.3)	37 (20.1)	45 (10.6)	<0.001

All participants were interviewed for dyspepsia symptoms and their severity over a period of 6 months preceding the survey using questionnaires set for the evaluation of the DSSI score [26], the GDSS [27], and GOS Scale [28]. The feeling of stomach burn was the most frequently reported symptom (212 out of 425, 49.9%). About 56.0% of subjects reported at least one dyspepsia symptom. The prevalence of the dyspepsia syndrome as defined by the Rome IV criteria was estimated 52.2% [95%CI: 47.4; 57.1]. Dyspepsia symptoms were reportedly occurring almost daily in about 25.2% of participants. In 26.1% of cases, the dyspepsia had been interfering with usual social activities, leading to a loss of one to seven days of activities in 15.8% of cases. Though a dyspepsia syndrome had been evoked by a health care worker in 56.0% of subjects, no one had ever been specifically tested or treated for *H*. *pylori* infection. Moreover, almost all the respondents (98.6%) were unaware of *H*. *pylori*. The proportion of participants with different dyspepsia symptoms was not significantly different between *H*. *pylori*-seropositive and -seronegative subjects (p>0.05) (**Tables 6 and 7**).

**Table 6:** Baseline clinical characteristics of study participants (n = 425).

Dyspepsia symptoms and their severity	Anti-*H*. *pylori* serology		p-value
	Seropositive	Seronegative	
	n (%) or median (IQR)	n (%) or median (IQR)	
**Symptoms (ref.: no such symptom)**			
Burping or belching	71 (49.3)	73 (50.7)	0.507
Bloating	49 (48.0)	53 (52.0)	0.820
Feeling full for very long time after meals	48 (50.5)	47 (49.5)	0.443
Inability to finish normal-sized meals	32 (42.1)	44 (57.9)	0.340
Abdominal discomfort, without pain, after meals	45 (49.5)	46 (50.5)	0.606
Abdominal distension	34 (43.6)	44 (56.4)	0.497
Nausea before meals	60 (52.2)	55 (47.8)	0.199
Nausea after meals	63 (50.4)	62 (49.6)	0.373
Nausea when waking up in the morning	49 (56.3)	38 (43.7)	0.053
Retching	35 (52.2)	32 (47.8)	0.355
Vomiting	31 (50.0)	31 (50.0)	0.616
Burping with bitter tasting fluid in throat	53 (46.9)	60 (53.1)	0.969
Regurgitation of bitter fluid into your mouth during the day	34 (43.6)	44 (56.4)	0.497
Regurgitation or reflux at night	31 (50.0)	31 (50.0)	0.616
Heartburn feeling	54 (46.2)	63 (53.8)	0.818
Burning feeling in the stomach	102 (48.1)	110 (51.9)	0.664
Abdominal ache or pain right after meals	40 (47.1)	45 (52.9)	1.000
Abdominal pain before meals or when hungry	44 (45.8)	52 (54.2)	0.785
Abdominal pain at night	30 (44.8)	37 (55.2)	0.684
Presence of at least one of the dyspeptic symptoms	112 (47.1)	126 (52.9)	1.000
History of a dyspeptic syndrome evoked by a health care giver	108 (48.2)	116 (51.8)	0.614
Dyspeptic syndrome as per Rome IV diagnostic criteria	108 (48.6)	114 (51.4)	0.492
**Severity of dyspepsia**			
GOS scale: No problem	92 (50.5)	90 (49.5)	-
GOS scale: Minimal problem (Ref.: No problem)	30 (56.6)	23 (43.4)	0.438
GOS scale: Mild problem (Ref.: No problem)	17 (41.5)	24 (58.5)	0.295
GOS scale: Moderate problem (Ref.: No problem)	15 (44.1)	19 (55.9)	0.492
GOS scale: Moderately severe problem (Ref.: No problem)	19 (51.4)	18 (48.6)	0.929
GOS scale: Severe problem (Ref.: No problem)	14 (56.0)	11 (44.0)	0.610
GOS scale: Very severe problem (Ref.: No problem)	4 (33.3)	8 (66.7)	0.256
DSSI scale: Dysmotility-like item	2 (10)	2.5 (11)	0.184
DSSI scale: Reflux-like item	2 (7)	2 (7)	0.273
DSSI scale: Ulcer-like item	0 (2)	0 (2)	0.483
DSSI scale: Overall DSSI item	1 (3)	1 (3)	0.159
DSSI scale: Total DSSI	1.3 (6)	1.8 (7)	0.254
GDSS score	1 (6)	1 (6)	0.118

**Table 7:** Characterization of the dyspepsia in study participants.

Characteristics	n	%
**Frequency of onset of dyspepsia symptoms during the last 6 months**		
Never or on less than 1 day a month	199	46.8
On only 1 or 2 days a month	88	20.7
About 1 day a month	30	7.1
About 1 day a week	0	0.0
About 50% of the days of the month	1	0.2
Almost every day of the month	107	25.2
**Impact of dyspepsia symptoms on social activities**		
Participants without any dyspeptic symptom	187	44.0
Participants with symptoms that do not interfere with normal social activities (e.g., sleeping)	73	17.2
Participants with symptoms that interfere a few times with normal social activities	111	26.1
Participant with symptoms that interact regularly with normal social activities	54	12.7
**Estimated number of activity days lost due to the occurrence of dyspepsia symptoms**		
0 day	327	76.9
1–7 days	67	15.8
More than 7 days	31	7.3
**Medical prescription received from a health care provider to treat dyspepsia**		
Not once	374	88.0
At least once	51	12.0
**Self-administration of a treatments against dyspeptic symptoms**		
Not once	306	72.0
At least once	119	28.0
**History of a medical diagnosis of the dyspeptic syndrome in the past**		
Present	224	52.7
Absent	201	47.3
**History of *H*. *pylori* test undertaken in the past**		
Not once	425	100.0
At least once	0	0.0
**History of *H*. *pylori* eradication treatment taken in the past**		
Not once	425	100.0
At least once	0	0.0
**Awareness of *H*. *pylori* infection**		
Participants who had already heard of *H*. *pylori*	6	1.4
Participants who had never heard of *H*. *pylori*	419	98.6

The final logistic regression model indicated that the age of participants, crowding of households, and quality of hand hygiene were independently associated with the *H*. *pylori*-seropositivity (**Table 8**). Hence, for every year increase in the age of participants, the likelihood of *H*. *pylori*-seropositivity increased by one unit (adjusted odds ratio [aOR] = 1.0 [95%CI: 1.0; 1.1]; p<0.001). Subjects living in overcrowded households (i.e., where more than two residents share the same sleeping room) were more than three times associated with an *H*. *pylori*-seropositive status compared to those living in less crowded households (aOR = 3.2 [95%CI: 2.0; 5.1]; p<0.001). Individuals with a poor hand hygiene were four times and half more *H*. *pylori*-seropositive than those with good hand hygiene (aOR = 4.5 [95%CI: 2.0; 11.4]; p = 0.001). No other putative predictor or clinical feature (dyspeptic symptoms and their severity score) was significantly associated to the *H*. *pylori*-seropositive status in the model.

**Table 8:** Logistic regression models detecting factors that predict the *H*. *pylori* seropositivity in the study population*.

Factor	Initial multi-variable model		Final multi-variable model	
	cOR [CI95%]	p-value	aOR [CI95%]	p-value
Male gender (Ref: Female)	0.6 [0.4; 1.1]	0.121		
Age (years old)	>1.0 [>1.0; 1.1]	<0.001	>1.0 [>1.0; 1.1]	<0.001
Single (Ref: Married/divorced/widower or widow)	1.2 [0.6; 2.4]	0.606		
Christian religion (Ref: Non-Christian religion)	0.5 [0.1; 1.6]	0.217		
College/University education (Ref: Elementary education)	2.3 [0.8; 6.8]	0.128		
High school education (Ref: Elementary education)	1.8 [0.9; 3.5]	0.078		
Not holding a regular profession (Ref: Holding a profession)	0.6 [0.3; 0.9]	0.033		
Overcrowded households (Ref: Less crowded households)	2.2 [1.7; 3.0]	<0.001	3.2 [2.0; 5.1]	<0.001
Duration of residence in the area (years)	1.0 [1.0; 1.0]	0.421		
Socioeconomic category 2 "Less poor" (Ref: Category 1 "Most poor")	0.8 [0.5; 1.4]	0.467		
Tape water as source for daily drinking water (Ref: Well water)	1.0 [0.0; 9.0]	0.995	0.7 [0.0; 6.8]	0.792
Use of toilet facilities shared between several families in household (Ref: Private toilets)	0.9 [0.4; 1.9]	0.831		
Non-optimal hand hygiene (Ref: Optimal hand hygiene)	4.1 [1.6; 11.7]	0.005	4.5 [2.0; 11.4]	0.001
Knowledge of *H*. *pylori* (Ref: lack of knowledge of *H*. *pylori*)	0.8 [0.1; 4.2]	0. 739		
Burping or belching (Ref: No such symptom)	1.4 [0.6; 3.0]	0.423		
Bloating (Ref: No such symptom)	1.6 [0.8; 3.4]	0.201		
Feeling full for very long time after meals (Ref: No such symptom)	1.5 [0.7; 3.6]	0.322		
Inability to finish normal-sized meals (Ref: No such symptom)	0.7 [0.3; 1.6]	0.369		
Abdominal discomfort, without pain, after meals (Ref: No such symptom)	1.2 [0.6; 2.5]	0.684		
Abdominal distension (Ref: No such symptom)	0.5 [0.2; 1.2]	0.127		
Nausea before meals (Ref: No such symptom)	1.4 [0.6; 3.1]	0.447		
Nausea after meals (Ref: No such symptom)	1.2 [0.6; 2.7]	0.588		
Nausea when waking up in the morning (Ref: No such symptom)	1.8 [0.8; 4.2]	0.152	1.3 [0.8; 2.3]	0.323
Retching (Ref: No such symptom)	1.1 [0.5; 2.6]	0.848		
Vomiting (Ref: No such symptom)	0.8 [0.3; 2.0]	0.652		
Burping with bitter tasting fluid in throat (Ref: No such symptom)	1.2 [0.5; 2.6]	0.720		
Regurgitation of bitter fluid into your mouth during the day (Ref: No such symptom)	1.1 [0.4; 2.5]	0.909		
Heartburn feeling (Ref: No such symptom)	0.7 [0.3; 1.5]	0.333		
Burning feeling in the stomach (Ref: No such symptom)	0.6 [0.1; 2.8]	0.518		
Abdominal ache or pain right after meals (Ref: No such symptom)	0.5 [0.3; 1.1]	0.101		
Abdominal pain at night (Ref: No such symptom)	2.6 [1.1; 6.3]	0.029		
Abdominal pain before meals or when hungry (Ref: No such symptom)	1.0 [0.5; 2.1]	1.000		
Regurgitation or reflux at night (Ref: No such symptom)	1.0 [0.6; 1.8]	0.502		
Minimal GOS problem (Ref.: No problem)	0.8 [0.3; 2.7]	0.747		
Mild GOS problem (Ref.: No problem)	1.4 [0.4; 4.2]	0.596		
Moderate GOS problem (Ref.: No problem)	1.7 [0.5; 5.7]	0.375		
Moderately severe GOS problem of dyspepsia (Ref.: No problem)	1.6 [0.4; 5.8]	0.478		
Severe GOS problem (Ref.: No problem)	0.8 [0.2; 3.3]	0.723		
Very severe GOS problem (Ref.: No problem)	1.1 [0.2; 7.3]	0.940		
DSSI scale	0.9 [0.8; 1.1]	0.433		
GDSS score	1.1 [1.0; 1.3]	0.146		
Presence of at least one of the dyspeptic symptoms (Ref: No such symptom)	0.4 [0.1; 2.2]	0.317		
History of a dyspeptic syndrome evoked by a health care giver (Ref: No such symptom)	1.0 [0.3; 3.7]	0.960		
Dyspeptic syndrome as per Rome IV diagnostic criteria (Ref: No Syndrome)	1.6 [0.2; 13.9]	0.659		

(*) cOR, crude odds ratio; aOR, adjusted odds ratio

## Discussion

To our knowledge, this is the first study that develops an ELISA specifically tailored for mass screening of *H*. *pylori* infection in Africa and which is then applied to a real-world epidemiological survey conducted in African setting. This indirect ELISA system, termed HpAfr-ELISA, was conceived for the detection of anti*-H*. *pylori* IgG antibodies directed against whole-cell antigens of strains from *hpAfrica1* and *hpNEAfrica*, the two main genetic populations of *H*. *pylori* species that infect Africans [21,31,32]. Initially, we assessed the diagnostic performance and the robustness of the assay by using a training set of samples previously collected from patients who had been tested with traditional invasive methods (i.e., histology, bacterial culture, and rapid urease test) and whose *H*. *pylori* infection status was known a priori. Then, we performed a cross-validation of this performance through samples from a community-based survey. Hence, the novel assay indicated high diagnostic performance on both the training set and the cross-validation set with sensitivity and specificity being >80%. Furthermore, the system provided consistent results in intra-observer for both qualitative (i.e., detecting *H*. *pylori*-seropositive or -seronegative status) and quantitative (i.e., anti*-H*. *pylori* IgG levels) outcomes. The performance of the HpAfr-ELISA was similar to that reported with assays tailored for other specific populations [50–55]. The needs for adjusting ELISA for *H*. *pylori* to specific populations has been raised by consistent evidence indicating that commercial ELISA kits with excellent performance in populations where they are originated from, demonstrate poorer performance when used in other regions [6,9–13]. In Africa, a survey using a commercial ELISA kit optimized at high performance in the United States (US) reported a very low sensitivity (50%) in comparison to invasive tests (e.g., culture, histology, and rapid urease test) in Egypt [56]. Inconsistent outcomes noted with ELISA kits throughout different regions likely indicate varied immunogenic properties in different population which are possibly sustained by genetic variations of *H*. *pylori* from parts of the world used as a source of antigen for the kits [9–13,57]. To adapt ELISA systems to a specific regions, local *H*. *pylori* strains could be used for preparing antigens rather than foreign strains [58]. Alternatively, common antigens shared globally by strains or strains pooled from different regions could be used [57,58]. Furthermore, best cut-off-values of antibody titers should be established and validated based on local epidemiology rather than values recommended by the assay’s manufacturer [58]. In terms of antigens, whole-cell antigens covering a broad range of bacterial antigens would provide more immunogenicity than isolated antigenic components [17]. The assay developed in the current study not only used local strains but also pooled strains from most representative genetic populations of Africa and applied whole-cell antigens rather than a specific single antigen. This may explain the high performance reached with the new assay. These results indicate that the HpAfr-ELISA assay is probably a useful tool for assessing accurately the *H*. *pylori* infection in African setting, notably in the DRC. Obviously, *H*. *pylori* serological tests detect individual who had been exposed to the pathogen including past infections cases. Consequently, they probably can be more useful as screening tools for populations than as diagnostic tools for individual diagnosis. As such, during strategies for the management of *H*. *pylori* like the “test-and-treat” approach [2,59], these tests would be helpful for filtering the vast majority of uninfected patients to allow focusing available resources on the most likely infected cases. Moreover, serological tests with their relative low costs and high-throughput capabilities can also be useful for evaluating and monitoring the disease burden in populations over periods of time. To further illustrate the usefulness of HpAfr-ELISA, we applied the assay for estimating the true prevalence of *H*. *pylori* infection and for identifying factors that expose to *H*. *pylori* in a community from the DRC.

Estimating the true prevalence of a disease constitute an important part of public health efforts that can help define efficient health policies and optimally allocating available resources. Bayesian methods applied in this study make it possible to estimate accurately the true prevalence in the absence of a perfect gold standard test [42,43,60]. The true prevalence of *H*. *pylori* in the study population was thus estimated 53.8% with a 95%CrI ranging from 42.8 to 62.7%. At the time of this publication, there was no study reporting the prevalence of *H*. *pylori* infection at community level within the DRC. Although consistently supporting a high prevalence of *H*. *pylori* in DRC, previous reports had only involved patients recruited in hospitals and are likely not faithfully reflecting the epidemiological situation in the general population of respective study areas [61–66]. Likewise, other observations also suggest a high prevalence in neighboring countries [67–70]. Furthermore, recent global meta-analyses of community-based studies indicated that such epidemiological situation might be present in most emerging and developing countries, especially in Africa, in contrast to developed countries where the *H*. *pylori* prevalence could be lower [3,4]. The high prevalence of *H*. *pylori* probably testify a persistent bacterial transmission coupled with limited access to adequate diagnosis and treatment of the infection in the studied community [71]. It is likely that the majority of seropositive subjects had chronic *H*. *pylori* infection, a condition associated with severe complications such as peptic ulcers and gastric cancers [2,72]. We identified an increased age as significantly predicting the *H*. *pylori*-seropositive status in this study. The link between *H*. *pylori* and age is widely discussed in previous studies [73–75]. Globally, in developed countries where the *H*. *pylori* prevalence seems to be decreasing during the last two decades, the link with age is likely due to the effect of birth cohorts of earlier generations that had been more exposed in the past to favoring factors such as poor sanitation [76]. In developing countries, oppositely, the prevalence of *H*. *pylori* infection seems to have remained stable over years [4]. Thus, the increase of *H*. *pylori* infections with growing age in these countries might result from an accumulation of infected but untreated people over time and in conditions persistently promoting the infection transmission. Long-term follow-up studies are however warranted to evaluate this hypothesis. Given the potential accumulation of infections with age, elders should attract maximum attention of health policy makers as they are additionally the most prone to severe *H*. *pylori*-related complications such as gastric cancers and peptic ulcer diseases with significantly higher mortality [76,77]. Beside the effect of age, life in overcrowded households and poor hand hygiene were also associated with the *H*. *pylori*-seropositive status. This suggests that conditions with low hygiene and precarious sanitation might be favoring *H*. *pylori* infections or perpetuating the human-to-human transmission of the bacteria in the study population. These observations are consistent with the global consensus on the epidemiology and the most likely transmission routes of *H*. *pylori* (e.g., oro-fecal and oro-oral routes) [78–80]. Therefore, enhanced efforts for improving individual hygiene are required to reduce the burden of *H*. *pylori* infection locally. Investigations on the main transmission routes and the age of acquisition should be undergone for strengthening the basis for effective prevention strategies.

This study comprised several limitations. The community-based survey included only adults from a specific location in the country. In addition, even though the serological test was created using strains of genetic populations known as colonizing most of the African continent, it was only tested against samples from DRC. Strains used for preparing the assay did not include those from *hpAfrica2*, a very divergent *H*. *pylori* population colonizing individuals from a specific ethnic of hunter-gatherers in southern Africa known as the San population [21,31,32]. Similarly, the assay did not take into account bacterial populations that had been introduced to Africa in recent history (e.g., *hpEurope* and *hpAsia2*) [20,21]. The generalizability of outcomes would thus need to be interpreted with caution. This survey was based on interviews and cannot be spared from memory bias that this entails. As an indirect diagnostic method, the assay developed in this study is prone to performance errors and misclassifications of samples. To reduce the possibility of these errors, we designed the assay to account for the local antigenicity background. We additionally used Bayesian methods to control statistical uncertainties in observations and to provide more accurate estimates despite the absence of a perfect gold standard test [42,43,60]. Though the validity of the assay could be shown, the assessment processed only in intra-observer without any external validation. Furthermore, the possibility of cross-reactivity between the assay and heterologous species or other host-related pathophysiological conditions could not be investigated. The assay remains thus perfectible and open to further improvements. The seropositivity to the HpAfr-ELISA assay did not correlate significantly with symptoms in this study. Though this is a general issue for serological tests of *H*. *pylori* infection, it can limit their utility for individual diagnosis [81]. Further evidence on the usefulness and the cost-effectiveness of this new kit should be provided in future studies that should include a comparative analysis against commercially available ELISA kits or other non-invasive tests of *H*. *pylori* (e.g., stool antigen test and UBT). The logistic regression models fitted to identify factors associated with the exposition to *H*. *pylori* in the community did not take the test sensitivity and specificity into account, but we acknowledge that the practical feasibility of this is not as easy as it seems. Finally, despite the high performance achieved by the assay, the Bayesian methods applied to assess the assay tool are still less commonly used in the medical field and this unfamiliarity may be seen also as a limitation for this study.

Notwithstanding the above limitations, this study has the merit of having successfully developed and validated the first ELISA specifically tailored for screening *H*. *pylori* infection in Africa. We believe that this assay is robust and provides reproducible outcomes with high diagnostic performance. Bayesian modeling enabled estimating a high latent true prevalence of *H*. *pylori* infection in the studied population that requires being addressed with appropriate public health strategies. Factors likely favoring *H*. *pylori* infection in the study area, i.e., growing age, life in overcrowded households, and poor hand hygiene, should specifically be targeted by management and mitigation strategies against the infection. Future explorations are warranted to further improve the novel ELISA system and to make it accessible for the needs of practical use. Operational research should be further implemented to extend our understanding of the epidemiology of *H*. *pylori* and to address this prevalent infection programmatically and efficiently in the study area and globally in Africa.

## Supporting information

S1 Fig Neighbor-joining phylogenetic tree inferring the genetic populations of H. pylori strains used for the HpAfr-ELISA along with reference strains. (PDF)

S2 Fig Electrophoretic profile of Whole-cell antigen proteins used for the HpAfr-ELISA. (PDF)

S1 Table STROBE Statement–Checklist of items that are included in the reports. (PDF)

S2 Table Characteristics of the training samples used for the HpAfr-ELISA system (n = 60). (PDF)

S1 File Bayesian statistical modeling. (PDF)
