# Supplementary material for: Assessment of the diagnostic accuracy and relevance of a novel ELISA system developed for seroepidemiologic surveys of Helicobacter pylori infection in African settings
Source: PLoS Negl Trop Dis. 2021 Sep 9;15(9):e0009763. doi: 10.1371/journal.pntd.0009763 (PMC8455143; doi:10.1371/journal.pntd.0009763)
Supplement: S1 Fig — (PDF) [file pntd.0009763.s001.pdf]

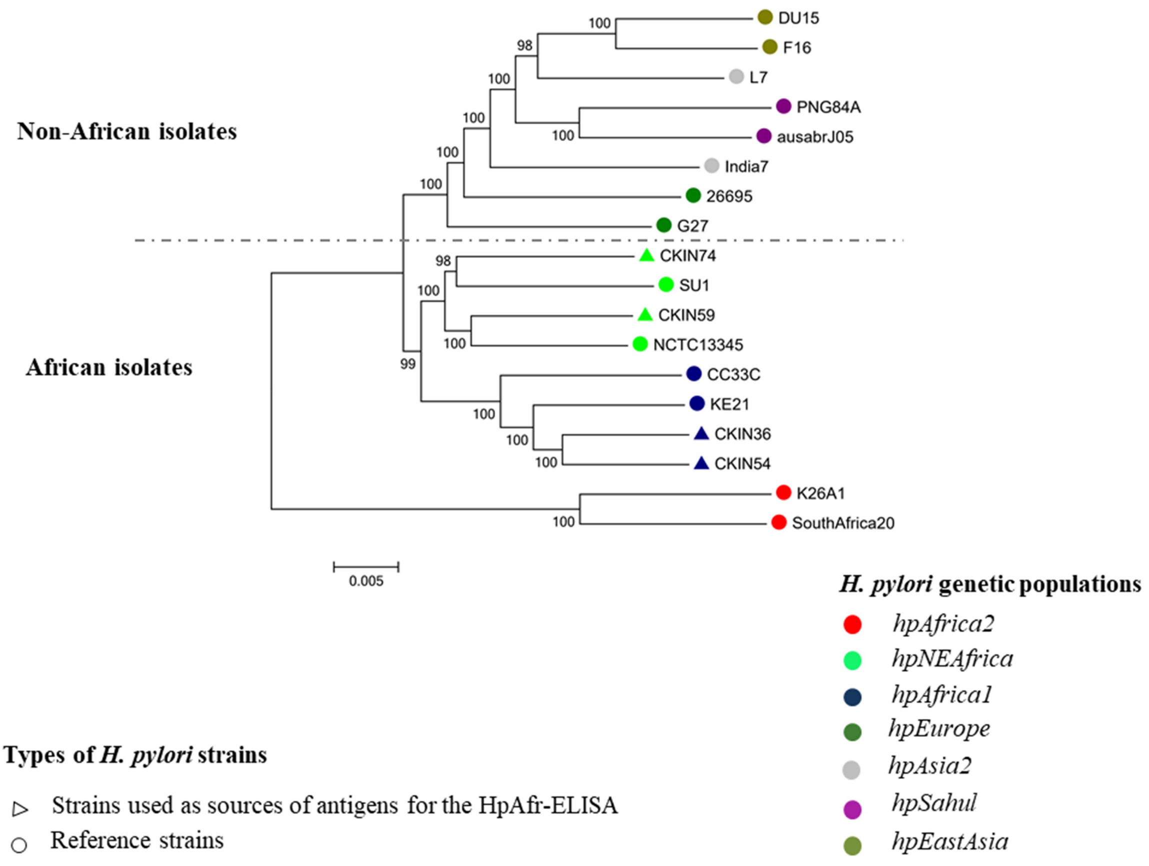

S1 Fig. Neighbor-joining phylogenetic tree inferring the genetic populations of H. pylori strains used for the HpAfr-ELISA along with reference strains.

This plot represent the molecular phylogeny of *H. pylori* strains used for developing the HpAfr-ELISA (4 isolates) and representative strains from different genetic populations existing in the species and arising from outside Africa (14 isolates). This neighbor-joining tree was estimated by Maximum Likelihood methods with 1000 boutstrap replications and based on concatenated nucleic acid sequences of 609 orthologous core genes (total length 589,770 bp) predicted by Prokka [1] and Roary [2] from genome sequences of all *H. pylori* strains of the dataset. Each *H. pylori* genetic population is assigned to a specific color on the phylogenetic tree. Strains used for the ELISA are labelled with triangle markers while circle markers are used for indicating reference strains. This figure indicates that *H. pylori* CKIN74 and CKIN59 were assigned to the clade of *hpNEAfrica* while CKIN36 and CKIN54 were assigned to *hpAfrica1,* the two main populations colonizing Africa [3-5]. Strains form the *hpAfrica2* population which have evolved in isolation for many millennia in people of San ethnicity [5,6], were not included in the assay.

Reference

1. Seemann T. Prokka: rapid prokaryotic genome annotation. Bioinformatics. 2014;30(14):2068-9.
2. Page AJ, Cummins CA, Hunt M, Wong VK, Reuter S, Holden MT, et al. Roary: rapid large-scale prokaryote pan genome analysis. Bioinformatics. 2015;31(22):3691-3.
3. Nell S, Eibach D, Montano V, Maady A, Nkwescheu A, Siri J, et al. Recent acquisition of Helicobacter pylori by Baka pygmies. PLoS genetics. 2013;9(9):e1003775.
4. Kabamba ET, Tuan VP, Yamaoka Y. Genetic populations and virulence factors of Helicobacter pylori. Infection, Genetics and Evolution. 2018;60:109-16.
5. Moodley Y, Linz B, Bond RP, Nieuwoudt M, Soodyall H, Schlebusch CM, et al. Age of the association between Helicobacter pylori and man. PLoS pathogens. 2012;8(5):e1002693. Epub 2012/05/17. doi: 10.1371/journal.ppat.1002693. PubMed PMID: 22589724; PubMed Central PMCID: PMC3349757.
6. Montano V, Didelot X, Foll M, Linz B, Reinhardt R, Suerbaum S, et al. Worldwide population structure, long-term demography, and local adaptation of Helicobacter pylori. Genetics. 2015;200(3):947-63.
