# Supplementary material for: Assessment of the diagnostic accuracy and relevance of a novel ELISA system developed for seroepidemiologic surveys of Helicobacter pylori infection in African settings
Source: PLoS Negl Trop Dis. 2021 Sep 9;15(9):e0009763. doi: 10.1371/journal.pntd.0009763 (PMC8455143; doi:10.1371/journal.pntd.0009763)
Supplement: S2 Fig — (PDF) [file pntd.0009763.s002.pdf]

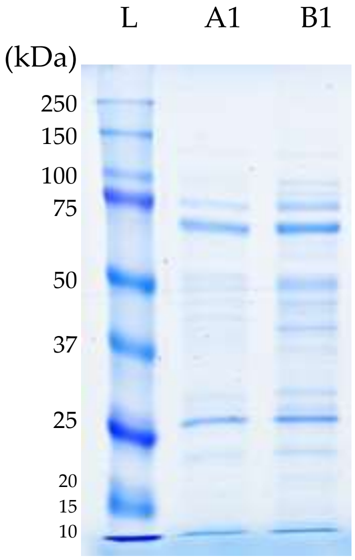

S2 Fig. Electrophoretic profile of Whole-cell antigen proteins used for the HpAfr-ELISA

This figure displays the gel obtained after the electrophoresis for separating whole-cell antigens that were used for coating the HpAfr-ELISA system. This is a CBB-stained 8% sodium dodecyl sulfate polyacrylamide gel (SDS-PAGE) gel that comprised 20 μg of proteins loaded per well. The protein standards (Precision Plus Protein™ Standards, Bio-Rad) were loaded in the well L. Lanes A1-B1 represent pooled cell lysates of strains from *hpNEAfrica* (A1) and *hpAfrica1* (B1) after protein dialysis.
