# Supplementary material for: Assessment of the diagnostic accuracy and relevance of a novel ELISA system developed for seroepidemiologic surveys of Helicobacter pylori infection in African settings
Source: PLoS Negl Trop Dis. 2021 Sep 9;15(9):e0009763. doi: 10.1371/journal.pntd.0009763 (PMC8455143; doi:10.1371/journal.pntd.0009763)
Supplement: S2 Table — (PDF) [file pntd.0009763.s004.pdf]

S2 Table. Characteristics of the training samples used for the HpAfr-ELISA system (n = 60)*

ID	Average anti-*H. pylori* IgG	RUT	Histology	Culture	*H. pylori* status
1	14.4	Negative	Negative	Negative	Negative
2	15.5	Negative	Negative	Negative	Negative
3	20.0	Negative	Negative	Negative	Negative
4	7.9	Negative	Negative	Negative	Negative
5	11.2	Negative	Negative	Negative	Negative
6	7.6	Negative	Negative	Negative	Negative
7	6.7	Negative	Negative	Negative	Negative
8	8.4	Negative	Negative	Negative	Negative
9	20.1	Negative	Negative	Negative	Negative
10	5.3	Negative	Negative	Negative	Negative
11	3.4	Negative	Negative	Negative	Negative
12	13.3	Negative	Negative	Negative	Negative
13	9.7	Negative	Negative	Negative	Negative
14	14.9	Negative	Negative	Negative	Negative
15	10.0	Negative	Negative	Negative	Negative
16	17.4	Negative	Negative	Negative	Negative
17	30.8	Negative	Negative	Negative	Negative
18	5.9	Negative	Negative	Negative	Negative
19	6.9	Negative	Negative	Negative	Negative
20	10.7	Negative	Negative	Negative	Negative
21	7.4	Negative	Negative	Negative	Negative
22	8.5	Negative	Negative	Negative	Negative
23	13.9	Negative	Negative	Negative	Negative
24	24.6	Negative	Negative	Negative	Negative
25	17.4	Negative	Negative	Negative	Negative
26	22.2	Negative	Negative	Negative	Negative
27	12.6	Negative	Negative	Negative	Negative
28	12.9	Negative	Negative	Negative	Negative
29	13.2	Negative	Negative	Negative	Negative
30	9.0	Negative	Negative	Negative	Negative
31	31.2	Negative	Negative	Positive	Positive
32	291.2	Positive	Positive	Positive	Positive
33	204.8	Negative	Positive	Positive	Positive
34	180.7	Positive	Positive	Positive	Positive
35	83.4	Positive	Negative	Positive	Positive
36	40.3	Positive	Positive	Positive	Positive
37	30.1	Positive	Positive	Positive	Positive
38	82.9	Positive	Positive	Negative	Positive
39	59.9	Positive	Negative	Positive	Positive
40	28.5	Positive	Positive	Positive	Positive
41	111.1	Positive	Positive	Positive	Positive

42	130.6	Positive	Negative	Negative	Positive
43	517.3	Positive	Negative	Positive	Positive
44	248.5	Positive	Positive	Positive	Positive
45	48.3	Positive	Positive	Positive	Positive
46	28.6	Positive	Positive	Positive	Positive
47	36.9	Positive	Positive	Positive	Positive
48	497	Positive	Negative	Positive	Positive
49	57	Positive	Positive	Positive	Positive
50	25.1	Positive	Positive	Positive	Positive
51	20.2	Positive	Positive	Negative	Positive
52	41.5	Positive	Negative	Positive	Positive
53	21.8	Positive	Positive	Negative	Positive
54	34.9	Positive	Positive	Negative	Positive
55	16.5	Positive	Positive	Positive	Positive
56	23.5	Positive	Positive	Positive	Positive
57	51.9	Positive	Negative	Positive	Positive
58	43.6	Positive	Negative	Positive	Positive
59	46.4	Positive	Negative	Positive	Positive
60	20.3	Positive	Positive	Positive	Positive

(*) RUT: rapid urease test ; Histology: standard histological examination with Giemsa staining and immunohistochemistry with primary antibody against whole cell H. pylori antigens, Western-type CagA protein, and SouthEast Asian-type CagA protein; Culture: bacterial culture using *Helicobacter* Selective plates in microaerophilic conditions [1-3].

References

1. Nell S, Eibach D, Montano V, Maady A, Nkwescheu A, Siri J, et al. Recent acquisition of Helicobacter pylori by Baka pygmies. PLoS genetics. 2013;9(9):e1003775.
2. Shiota S, Cruz M, Abreu JAJ, Mitsui T, Terao H, Disla M, et al. Virulence genes of Helicobacter pylori in the Dominican Republic. Journal of medical microbiology. 2014;63(Pt 9):1189.
3. Mwangi C, Njoroge S, Tshibangu-Kabamba E, Moloo Z, Rajula A, Devani S, et al. Whole Genome Sequencing Reveals Virulence Potentials of Helicobacter pylori Strain KE21 Isolated from a Kenyan Patient with Gastric Signet Ring Cell Carcinoma. Toxins. 2020;12(9):556.
