# Supplementary material for: Assessment of the diagnostic accuracy and relevance of a novel ELISA system developed for seroepidemiologic surveys of Helicobacter pylori infection in African settings
Source: PLoS Negl Trop Dis. 2021 Sep 9;15(9):e0009763. doi: 10.1371/journal.pntd.0009763 (PMC8455143; doi:10.1371/journal.pntd.0009763)
Supplement: S1 File — (PDF) [file pntd.0009763.s005.pdf]

Bayesian statistical modeling

Bayesian inferential methods were used in the current study. We are thus describing different models that were assumed. For a more thorough coverage on the models fitted and the Bayesian methods, see Speybroeck N et al. [1], Kruschke JK et al. [2-5], McElreath R. [6], Makowski D et al. [7,8], and Gelman A et al. [9,10]. The analyses were performed in R environment using mainly the following packages:

```r
# main packages used
library(tidyverse)
library(prevalence)
library(brms)
library(bayestestR)
```

Bayesian modeling of the true prevalence of *H. pylori* based on stochastic sensitivity and specificity

Subjects were screened using the HpAfr-ELISA test that detects anti-*H. pylori* IgG antibodies for estimating the prevalence of *H. pylori* in the study population. The diagnostic performance of this newly developed test is unknown. However, it is well known that anti-*H. pylori* IgG antibodies can be present for years after the infection has been lost providing false positive results [11]. In addition, false negative results can arise from cross-reactions with other pathogens or other pathophysiological conditions [11]. This serological test presents thus possibility of misclassification errors and its sensitivity (SE) and specificity (SP) likely do not reach both 100%. Therefore, this test cannot be consider as a gold standard for the diagnosis of *H. pylori*. Applying such test will provide an apparent prevalence (AP) may include or exclude misclassified cases while the real prevalence remains unknown.

To correct estimates arising from this kind of imperfect tests, Rogan WJ and Gladen B [12] had developed an adjusted estimator of the "true" prevalence (TP) that accounts for the sensitivity and the specificity of the screening test : $\mathrm{TP} = \frac{\mathrm{SP+A\ \ -1}}{\mathrm{SE+SP-1}}$ [12]. Then, Speybroeck N et al. [13] developed a Bayesian framework for estimating the TP based on the Rogan & Gladen equation. This Bayesian method is particularly useful in situations of imperfect tests with unknown sensitivity and SP. Actually, instead of assuming fixed values, the Bayesian model allows assuming that the sensitivity and the specificity can take several possible values between two defined bounds and following a conditional mathematical distributions. Hence, using a stochastic algorithm (e.g. MCMC simulations), Bayesian methods make it possible to estimate the posterior probabilities that reflect accurately the TP as well as the SP, and sensitivity of the screening test in the study population. Based on this Bayesian framework, a straightforwad approch for implementing the Rogan & Gladen equation has been provided in R via the

prevalence Package call on JAGS (Just Another Gibbs Sampler) through the *rjags* package for performing MCMC simulations [14,15].

The true Hp prevalence could be thus estimated using the Bayesian model of Speybroeck N et al. [13]. Given, the apparent test results are represented by a vector $\boldsymbol{x} = (x_1, x_0)$, with x_1 and x_0 the number of subjects respectively testing positive and negative with the HpAfr-ELISA system. The vector $\boldsymbol{x}$ was assumed to be distributed according to a binomial distribution with parameters AP and n (the number of study subjects). The posterior probabilities were sampled following a Beta(1, 1) prior distribution for the TP, a Beta(29, 1) prior distribution for the SE, and a Beta(27, 3) prior distribution for the SP. The boundaries of the latter two distributions corresponded to the prior information obtained experimentally with the training set. The full model was of form:

```
## To model SE and SP as following a Beta distribution based on the results of the training study defined as prior
# Defining Beta distributions as priors
SE <- list(dist = "beta", alpha = 29, beta = 1) #(mean = 29/30)
SP <- list(dist = "beta", alpha = 27, beta = 3) #(mean = 27/30)
# Estimating the true prevalence with credible intervals
TP <- truePrev(x = 241, n = 425, nchains = 4, SE = SE, SP = SP) #(AP = 241/425)
```

```
TP@model # showing the model
```

```
model {
  x ~ dbin(AP, n)
  AP <- SE * TP + (1 - SP) * (1 - TP)
  SE ~ dbeta(29, 1)
  SP ~ dbeta(27, 3)
  TP ~ dbeta(1, 1)
}
```

```
TP # showing the result
```

```
     mean  median  mode   sd     2.5%   97.5%
TP   0.536  0.538  0.542  0.048  0.434  0.626
SE  0.966   0.976  0.994  0.033  0.875  0.999
SP  0.897   0.906  0.925  0.055  0.766  0.977

Multivariate BGR statistic = 1.0007
BGR values substantially above 1 indicate lack of convergence
```

```
# Ploting the model parameters
par(mfcol = c(3, 3))
densplot(TP, col = "red")
```

```
traceplot(TP, col = "blue")
autocorr.plot(TP, col = "green")
```

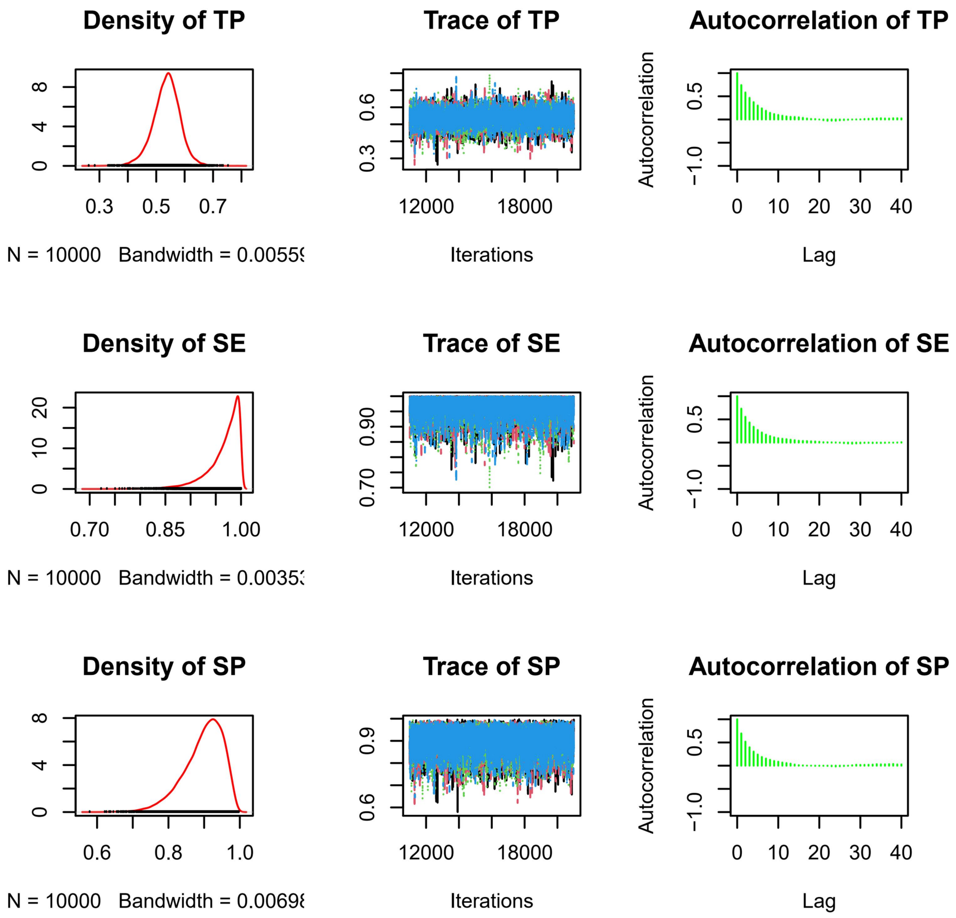

Additional functions inherits from *coda* package [16,17] were used to assess Gelman & Rubin's diagnostics for convergence of the model and to describe the MCMC output as Medians with a 95% credibility interval (95%-CrI) based on the **Highest Density Interval (HDI).**

```
# Highest Posterior Density Interval by coda package
HPDinterval(TP@mcmc$TP)
HPDinterval(TP@mcmc$SE)
HPDinterval(TP@mcmc$SP)
# Convergence diagnostics
TP@diagnostics # DIC and BGR (and bayesP)
gelman.diag(TP@mcmc$TP) #Gelman And Rubin's Convergence Diagnostic
gelman.diag(TP@mcmc$SE)
gelman.diag(TP@mcmc$SP)
```

Reference

1. Speybroeck N, Devleesschauwer B, Joseph L, Berkvens D. Misclassification errors in prevalence estimation: Bayesian handling with care. International journal of public health. 2013;58(5):791-5. Epub 2012/12/25. doi: 10.1007/s00038-012-0439-9. PubMed PMID: 23263198.
2. Kruschke J. Doing Bayesian data analysis: A tutorial with R, JAGS, and Stan: Academic Press; 2014.
3. Kruschke JK, Liddell TM. The Bayesian New Statistics: Hypothesis testing, estimation, meta-analysis, and power analysis from a Bayesian perspective. Psychonomic Bulletin & Review. 2018;25(1):178-206.
4. Kruschke JK, Aguinis H, Joo H. The time has come: Bayesian methods for data analysis in the organizational sciences. Organizational Research Methods. 2012;15(4):722-52.
5. Kruschke JK. What to believe: Bayesian methods for data analysis. Trends in cognitive sciences. 2010;14(7):293-300.
6. McElreath R. rethinking: Statistical Rethinking book package. R package version 1.391. 2014.
7. Makowski D, Ben-Shachar M, Lüdecke D. bayestestR: Describing Effects and their Uncertainty, Existence and Significance within the Bayesian Framework. Journal of Open Source Software. 2019;4(40):1541.
8. Makowski D, Ben-Shachar MS, Chen S, Lüdecke D. Indices of effect existence and significance in the Bayesian framework. Frontiers in Psychology. 2019;10:2767.
9. Gelman A, Hill J. Data analysis using regression and multilevel/hierarchical models: Cambridge university press; 2006.
10. Gelman A, Jakulin A, Pittau MG, Su Y-S. A weakly informative default prior distribution for logistic and other regression models. The Annals of Applied Statistics. 2008;2(4):1360-83.
11. Miernyk KM, Bruden DL, Bruce MG, McMahon BJ, Hennessy TW, Peters HV, et al. Dynamics of Helicobacter pylori-specific immunoglobulin G for 2 years after successful eradication of Helicobacter pylori infection in an American Indian and Alaska native population. Clin Vaccine Immunol. 2007;14(1):85-6.
12. Rogan WJ, Gladen B. Estimating prevalence from the results of a screening test. American journal of epidemiology. 1978;107(1):71-6.
13. Speybroeck N, Devleesschauwer B, Joseph L, Berkvens D. Misclassification errors in prevalence estimation: Bayesian handling with care. International journal of public health. 2013;58(5):791-5.
14. Devleesschauwer B, Torgerson PR, Charlier J, Levecke B, Praet N, Dorny P, et al. Package 'prevalence'. 2013.
15. Plummer M. JAGS: Just another Gibbs sampler. 2004.
16. Plummer M, Best N, Cowles K, Vines K. CODA: convergence diagnosis and output analysis for MCMC. R news. 2006;6(1):7-11.
17. Plummer M, Best N, Cowles K, Vines K. Package 'coda'. 2019.
